# Experiences of People with Kidney Disease Following the Implementation of the Compassionate Mindful Resilience Programme: Qualitative Findings from the COSMIC Study

**DOI:** 10.3390/healthcare11222926

**Published:** 2023-11-08

**Authors:** Anna Wilson, Clare McKeaveney, Claire Carswell, Karen Atkinson, Stephanie Burton, Clare McVeigh, Lisa Graham-Wisener, Erika Jääskeläinen, William Johnston, Daniel O’Rourke, Joanne Reid, Soham Rej, Ian Walsh, Helen Noble

**Affiliations:** 1 School of Nursing and Midwifery, Queen’s University Belfast, Belfast BT7 1NN, UK; c.mckeaveney@qub.ac.uk (C.M.); c.carswell@qub.ac.uk (C.C.); clare.mcveigh@qub.ac.uk (C.M.); helen.noble@qub.ac.uk (H.N.); 2 Department of Health Sciences, University of York, York YO10 5DD, UK; 3 MindfulnessUK, Taunton TA3 7QY, UK; karen.atkinson@mindfulnessuk.com; 4 School of Psychology, Queen’s University Belfast, Belfast BT7 1NN, UK; 5 Research Unit of Population Health, University of Oulu, 90570 Oulu, Finland; erika.jaaskelainen@oulu.fi; 6 Patient and Carer Education Partnership, School of Nursing and Midwifery, Queen’s University Belfast, Belfast BT7 1NN, UK; 7 Department of Psychiatry, McGill University, Montreal, QC H3A 0G4, Canada; soham.rej@mcgill.ca; 8 School of Medicine, Dentistry and Biomedical Sciences, Queen’s University Belfast, Belfast BT7 1NN, UK; 9 Knightsbridge Healthcare Group, Belfast BT9 5UB, UK; 10 Institute of Psychosexual Medicine, London W4 5YA, UK

**Keywords:** mindfulness, compassion, resilience, kidney disease, transplant, qualitative

## Abstract

Background: Kidney disease is a progressive, debilitating condition. Patients experience challenging physical and psychological symptoms and are at increased risk of anxiety, depression, and poor mental wellbeing. Access to specialist psychological or social support is limited, with inadequate provision of psychosocial support available across UK renal units. The COSMIC study (examining the acceptability and feasibility of the Compassionate Mindful Resilience programme for adult patients with chronic kidney disease) aimed to support a new service development project, in partnership with Kidney Care UK, by implementing the Compassionate Mindful Resilience (CMR) programme, developed by MindfulnessUK, and explore its feasibility for patients with stage 4 or 5 kidney disease and kidney transplant recipients. This paper reports on the qualitative exploratory work which examined the experiences of study participants, their adherence to practice, and the acceptability of the intervention. Method: Participants (n = 19) took part in semi-structured interviews, which were transcribed, coded, and thematically analysed. Results: Three themes (and nine subthemes) were reported: experiences of the CMR programme that facilitated subjective benefit, participants’ lived and shared experiences, and the practicalities of CMR programme participation. All participants reported that they found taking part in the CMR programme to be a beneficial experience. Conclusion: The CMR programme was found to be an acceptable intervention for people living with kidney disease and provided tools and techniques that support the mental health and wellbeing of this patient group. Further qualitative exploration into participant experience should be integrated within future trials of this intervention.

## 1. Introduction

Kidney disease is a progressive chronic condition, and patients living with end-stage kidney disease (ESKD), including those who have received a kidney transplant, experience difficult physical and psychological symptoms and diminished health-related quality of life [1]. Increased anxiety [2] and depression [3,4] are common, with one fifth of patients with chronic kidney disease diagnosed with depression [5] and an average of 38% (12% to 52%) diagnosed with anxiety [6]. Access to specialist psychological or social support is limited, with inadequate provision of psychosocial support available across UK renal units, as identified in the recent UK renal psychosocial workforce report carried out by the British Renal Society (recently reformed as the UK Kidney Association) and Kidney Care UK (KCUK) [7]. Since the onset of the COVID-19 pandemic, people with kidney disease have experienced additional risk factors due to their clinically extremely vulnerable (CEV) status [8] alongside restrictions which have severely impacted their mental health and wellbeing. Guidance for patients to ‘shield’ and socially distance from others was in place for an extended period of time for this patient group with many patients continuing to restrict face-to-face contact with others and reduce time outside the home [9,10,11]. The risk to health, increased social isolation and, diminished quality of life has led to an increase in psychological distress [12], therefore, it is essential that effective systems of psychosocial support are identified and implemented in a safe and accessible way.

Mindfulness interventions have increased in popularity and prevalence in recent years and are used as a stress-reduction technique applicable across a range of settings [13]. Benefits of mindfulness include reduced stress and anxiety, improved sleep, and an increase in resilience, which contribute to improved mental health and wellbeing [13]. The Mindfulness-Based Stress Reduction (MBSR) programme, was developed at the University of Massachusetts Medical Center by Professor Jon Kabat-Zinn as an eight-week programme intended to complement clinical treatment plans and support patient wellbeing [14]. The programme is considered the ‘gold-standard’, and focuses an individual’s attention on internal and external stimuli in order to remain in the present moment and reduce disruptive thoughts [15]. Mindfulness interventions such as the MBSR have been successfully implemented for patients living with a range of chronic conditions with positive outcomes [16,17], with increasing popularity of online programmes [18]. A number of studies examining mindfulness interventions have been undertaken within the kidney disease population [19,20,21,22,23,24,25]; however, there is a paucity of qualitative research examining mindfulness interventions which integrate compassion and resilience.

The COSMIC study aimed to support a new service development project in partnership with KCUK, the UK’s leading patient support charity, by implementing the four-session Compassionate Mindful Resilience (CMR) programme, developed by MindfulnessUK, for patients with stage 4 (severe kidney disease, estimated glomerular filtration rate (eGFR) 15–29 mL/min per 1 · 73 m2) or stage 5 (kidney failure, GFR below 15 mL/min per 1 · 73 m2) chronic kidney disease [26] or who have received a kidney transplant [27]. It is the first study to explore the feasibility and acceptability of this intervention for this patient cohort. This paper reports the exploratory qualitative work undertaken as part of the larger feasibility study, with the objective to examine the experiences of study participants after completing the CMR programme and their adherence to practice, alongside factors influencing the acceptability of the intervention.

### Intervention

The CMR course is delivered for two hours per week for four consecutive weeks, in an online group setting. The programme was developed by Karen Atkinson, executive committee member for the British Association of Mindfulness Based Approaches [28], and MindfulnessUK [29]. The CMR programme was developed in response to a need for easily accessible mindfulness and compassion practices, and, like the MBSR programme [14] and Brach’s compassionate approach to psychotherapy [30], draws on Buddhist and Yoga philosophy blended with current psychoeducational intervention practices. The CMR programme introduces teaching simple, evidence-based practices, skills, and techniques to help people manage stress, such as body scan, compassionate movement, meditation, and breathing practices with a specific focus on enhancing self-compassion and resilience. The four components of the programme are: Exploring Mindfulness and Compassion, Cultivating Emotional Intelligence, Developing Resilience, and Feeling Resourced and Connected. Home practices are provided and encouraged but are not an essential requirement.

The Mindfulness Teacher undertaking the delivery of the CMR programme must have completed a robust Mindfulness Teacher training pathway and CMR training with MindfulnessUK. The first CMR programme was delivered by HN, an experienced nephrology nurse and Mindfulness Teacher. This was at the request of KCUK who felt a healthcare professional with nephrology experience would be able to identify any difficulties faced by participants with kidney disease or any changes required. No changes were necessary. Dr Michele Kavanagh, a consultant clinical psychologist and Mindfulness Teacher, delivered subsequent sessions. Both have trained with MindfulnessUK in the delivery of the CMR programme.

## 2. Materials and Methods

### 2.1. Design

A qualitative descriptive investigation was utilised as part of the larger single-group multi-methods feasibility study, detailed in the study protocol [31]. The consolidated criteria for reporting qualitative research (COREQ) checklist has been used to report the qualitative paper (available as Supplementary Materials S1) [32].

### 2.2. Recruitment

Participants for the qualitative interviews were recruited from the pool of participants who had completed at least three out of the four sessions of the CMR programme (n = 65) as part of the wider feasibility study.

Eligibility Criteria for the feasibility study:

- Over 18 years old;
- Currently living in the UK;
- In stage 4 or 5 kidney disease or have received a kidney transplant;
- Capacity to provide informed consent;
- Not undergoing psychotherapy;
- The CMR programme may not be recommended for individuals experiencing severe anxiety, depression, mental illness, addiction, recent bereavement, or a traumatic life event.

Participants recruited to the feasibility study self-assessed their eligibility for the study during registration, and undertook a one-to-one assessment with the Mindfulness Teacher, utilising an assessment tool developed in consultation with MindfulnessUK and KCUK prior to commencing the CMR programme.

Participants who completed the four-session CMR programme were invited via email to take part in a one-to-one qualitative interview. Those who expressed interest were sent a participant information sheet providing details of the motivation and procedures of the qualitative investigation and researchers’ information and credentials, and a consent form via email to complete and return. A purposive sampling strategy was utilised to include a range of participants and representation from each of the eight CMR programme groups. Each group was made up of either nine or ten participants.

### 2.3. Sample Size

While there is no formal criteria for determining sample size in qualitative research, a sample of 12 participants is considered appropriate to saturate data [33]. A sample of 19 were included and data saturation reached [34].

### 2.4. Data Collection

The semi-structured interviews were informed by the RE-AIM QuEST framework which proposes open-ended questions across five dimensions of Reach, Effectiveness, Adoption, Implementation, and Maintenance to guide evaluation of the intervention [35]. The participant interview schedule is available as Supplementary Materials S2. Study participants took part in the CMR programme between June and October 2022, and interviews were conducted within two to seven weeks of programme completion. The one-to-one interviews were conducted via the Zoom online meeting platform [36], at a time and date to suit the participant, and were conducted by the Research Assistant AW and reviewed by the Chief Investigator HN. The duration of the interviews ranged from 20–60 minutes, 20 open-ended questions, including sub-questions and prompts, were asked; no repeat interviews or field notes were undertaken, and interview transcripts were not returned to participants prior to data analysis. Transcripts were pseudonymised prior to review by the wider research team.

### 2.5. Data Analysis

The semi-structured interviews were recorded, transcribed verbatim by a professional transcriber, and thematically analysed and managed using NVivo qualitative analysis software v.12 [37]. An inductive approach to thematic analysis was used to analyse the qualitative data collected [38] and followed a six-stage process: familiarization with data, generating initial codes, searching for themes, reviewing themes, defining and naming themes, and producing the reported themes [39]. Interviews were coded and analysed by the Research Assistant AW and Chief Investigator HN, who have wide experience in qualitative research methodology and have undertaken extensive interview training as part of their postgraduate study. The interviews were reviewed by the research team. Data saturation was reached at 19 interviews. We did not ask participants to provide feedback on findings.

## 3. Results

Of the 65 participants from the eight CMR groups invited to participate in the interview, 21 agreed to participate (32%). One participant subsequently withdrew due to personal circumstances, and one did not return a completed consent form. A total of 19 participants, representing seven out of the eight CMR programme groups, were interviewed.

The mean age of interview participants was 51.6, and most were female (n = 14, 73.7%). The majority of interview participants were post-transplant (n = 11, 57.9%), 21.1% (n = 4) were in stage 4 kidney disease, and 21.1% (n = 4) were in stage 5. Most interview participants were from an English, Welsh, Scottish, Northern Irish, or British background (n = 13, 68.4%). An overview of interview participant characteristics is available in Table 1.

Three key themes with nine subthemes were generated from the interviews (summarised in Table 2): experiences of the CMR programme that facilitated subjective benefit, participants’ lived and shared experiences, and the practicalities of CMR programme participation.

### 3.1. Theme 1: Experiences of the CMR Programme That Facilitated Subjective Benefit

This theme highlights previous experience of mindfulness and motivations to participate in the CMR programme, and how participants have integrated mindfulness techniques and practices and are continuing their mindfulness practice.

#### 3.1.1. Subtheme 1: Interest in Mindfulness and Previous Experience

Participants identified a range of motivating factors for participating in the mindfulness programme, with most stating that they were interested in the potential to support their own mental wellbeing through mindfulness practices.

*“It was an opportunity to help me become more at peace with myself and my thoughts.”*

CMR026 (Participant Identification Number)

Several participants responded that due to their ongoing condition, they were anticipating changes in their health and thought that undertaking the programme might provide tools and techniques to help navigate difficult periods and improve resilience during challenging situations.

*“I thought that it would be a good thing to do to try and build up some of the resilience that I used to have. Because at some point in the future, I will need to be strong again, and I know that at the moment I’m not. So if it was something that I could learn and a skill that I could have to take forward for when I need it most.”*

CMR058

Participants had a range of previous mindfulness experience, with some having been introduced to meditation and breathing exercises through yoga practice. Some had been offered talking therapies during the early stages of their kidney disease diagnosis. A small number had undertaken mindfulness courses through their workplace, but most had little or no previous experience.

*“I’ve done some yoga, and yoga nidra in particular, where you think your way around your body, which was similar to the compassionate body scan that we did at the first session. And many years ago, when my kidneys first failed, whilst I was still on dialysis, I had some counselling and part of that was learning breathing techniques. So I’ve done similar things, but nothing actually called mindfulness.”*

CMR058

#### 3.1.2. Subtheme 2: Integrating Mindfulness Using Techniques and Practices to Enhance Awareness and Compassion

Participants reflected on the benefits they have experienced since taking part in the programme and how they have integrated mindfulness practices into their lives. Participants expressed that mindfulness has helped them become more aware of their emotions, to accept that feelings will pass, and to identify their own strengths. All participants stated that participating in the CMR was a beneficial experience.

*“Mindfulness has really consolidated the fact that my feelings are valid. And that I can learn to understand them and ‘control them’. Meaning, my feelings and/or my thoughts are not necessarily… they are just that. They are things that pass through me.”*

CMR026

Participants identified that their ability to live ‘in the present moment’ had improved, and that they experienced a greater sense of calm and improved wellbeing when employing the skills that they learned from the programme.

*“I think just the general feeling of wellbeing. When you’re doing the breathing and deep into one of the mindfulness practices… how calm and relaxing that feels and how it helps steady everything. Because that feels so good. It makes you want to carry on learning about it and to be able to do it to the best that you can.”*

CMR058

Participants identified practices they found to be specifically beneficial for them, in particular the body scan practice which helped manage physical symptoms and supported better sleep, meditation practices which encourage compassionate thinking, and the gratitude practice which focuses on positive experiences.

*“The practices, I have been doing ones that I found beneficial. The one that I keep going back to is the body scan, because the likes of, with the anxiety I find it’s either in my chest, with tight chest or fast beating heart, or in my stomach, it goes into knots. And that helps me calm the physical sensations of that down, and then because of that, then mentally I am calmer as well.”*

CMR044

The majority of participants reported that taking part in the CMR programme had helped them to become more aware of their emotions. Participants reported an increased ability to manage negative and intrusive thoughts.

*“I think just stepping back from things and being able to control my thoughts a bit better. Not letting them run away with me too much. I won’t say it never happens still, but I’m aware that it’s happening and sometimes I can actually switch my thoughts onto something different.”*

CMR004

Participants connected with the theme of compassion within the CMR programme, both in feeling more compassionate towards others, including family and friends, and towards themselves, particularly in recognising the difficulties they had experienced during their kidney disease journey.

*“I think the course… gave permission to people to feel compassion for themselves. To recognise that what they have is real. This pain is real. And therefore, you are allowed, you have permission to seek help or ask for help or learn more or… you know, give yourself time.”*

CMR024

Many participants reported that the focus on compassion and self-compassion was one of the most meaningful elements of the programme, had improved their ability to self-empathise, and enabled them to be kinder to themselves, particularly when faced with challenges.

*“I really learned and understand what the statement means to be kind to yourself. And I grew to actually learn to love myself more. I really learned how to embrace me and… I think the compassion part was probably the… the best part of it for me. Be kind. Be kind to yourself. Be aware”*

CMR026

#### 3.1.3. Subtheme 3: Continuing Mindfulness Practice

Participants reflected on the ways that they have been continuing their mindfulness, with many participants dedicating time to engage with the practices or incorporating them into their daily routines.

*“It’s really switching off from what’s going on around me. I come down to my shed in the garden, where I’ve got my office. So I can shut out the world and that’s really good. And it’s making me do it. Because I’m thinking, I’ve got to do it. Not because I have to do it, but because I want to do it.”*

CMR051

While most participants indicated an intention to continue practicing mindfulness, many expressed an interest in an ongoing practice which they could engage with on a more regular basis.

*“I would love refreshers. You know like you go to see the consultant every six months or something, it would be lovely to have a wee quick session just to focus your mind. Even if it was away in the distance, you would know it’s coming. I think it keeps you mindful.”*

CMR037

Participants expressed an interest in meeting with group members, or other people living with kidney disease who had undertaken the CMR programme, to continue their mindfulness practice and create a supportive community.

*“If we were sort of pushed to have maybe monthly calls just to chat and catch up with each other, that’s possibly the way forward. But building a community around mindfulness and just… I mean maybe this is broader than the mindfulness course, but Kidney Care could do an awful lot by bringing people, patients, together.”*

CMR034

### 3.2. Theme 2: Participants’ Lived and Shared Experiences

This theme highlights the impact of kidney disease of participants’ mental wellbeing, the value of shared experiences with other patients, and the need for ongoing wellbeing support.

#### 3.2.1. Subtheme 1: Psychological Impact of Kidney Disease and Continuing Impact of COVID-19

Many participants suffered from poor mental wellbeing during their diagnosis and treatment for kidney disease, in particular anxiety around their poor health and its impact on their lives.

*“I’m struggling to acknowledge where I am in the process, I think. Very personally, if you like. So I’m having to ease back on some of the things I love doing, playing football and things like that. And I’ve found it really challenging to get my head around doing that.”*

CMR007

Participants identified that being diagnosed with kidney disease, and the subsequent treatment options and transplant, had been traumatic, and many were still struggling to come to terms with this experience.

*“I was diagnosed and then three weeks from diagnosis I was getting dialysed. So when it’s kind of traumatic that way, and you spend all that time on dialysis away from your family, that’s a struggle.”*

CMR044

While most participants who took part in the study had received a kidney transplant, many continued to experience complicated feelings around their transplant, the limitations it places on their lives, and the knowledge that the transplanted kidney will eventually fail and they will return to haemodialysis.

*“I have been transplanted for fourteen and a half years. So for some people, that is a really long time. And I am very grateful for that. But then obviously there are still negativities associated with that…I am still different from the regular person walking down the street in that I have a transplant.”*

CMR044

Many participants identified the continuing impact of COVID-19 on their lives, which has brought increased anxiety and isolation to a group of people who are already living with a complicated chronic condition.

*“I’m still feeling the effects of… well very much so feeling the effects of COVID. Because I’ve been shielding. And I’m still semi-shielding now, so it’s been about three years. And when I feel very vulnerable, I feel very… I feel like a quarter of the person I used to be.”*

CMR024

#### 3.2.2. Subtheme 2: Shared Experiences with Other Study Participants

Almost all participants interviewed enjoyed connecting with other people with shared experience of living with kidney disease, as they did not often have opportunities to interact with others experiencing the condition.

*“More than anything, just the fact that I met people, especially when we broke off into the groups. I was actually talking to somebody who had the same disease, who was feeling the same, and it was like finding… it was like winning the lottery.”*

CMR024

The experience of connecting with others enabled participants to feel supported by their peers, and created opportunities to discuss symptoms, treatment plans, and share complex emotions about living with chronic kidney disease.

*“I would say is that the benefit we got through talking with each other in the groups, was so beneficial. So beneficial. We need more of that. Absolutely more of that. Getting that connection. Getting an understanding. That feeling that you’re not on your own.”*

CMR024

Participants felt that utilising breakout rooms was crucial to support these conversations and enable peer support, and that providing space for discussion was a key component of the CMR programme.

*“People are stressed and people are suffering… I think mindfulness is very useful for them, but simplify it and give them a chance to chat amongst themselves. And I think trying to build a community is an important part of this.”*

CMR034

#### 3.2.3. Subtheme 3: Need for Wellbeing Support for People Living with Kidney Disease

Participants reflected that being diagnosed with, and undergoing treatment for kidney disease, can be a traumatic experience, and while medical intervention is the highest priority, participants felt that their mental wellbeing had been impacted and required more support.

*“You are dealing with something at that stage, that you know your kidney is going to eventually give up, very soon probably. How do you deal with that? You’ve got a major body part that keeps your body functioning, that doesn’t work. How do you cope with that in your mind? If you can be given a key to help that along, then yeah, for sure it’s going to benefit.”*

CMR043

Participants identified a lack of support for people living with kidney disease, and that early interventions such as a mindfulness programme could be beneficial to manage the increase in anxiety experienced by many patients during their diagnosis and treatment.

*“I felt as if I was falling at that stage, I was coming down one of them big circular slides. I just felt I was sliding and I had nothing to hold on to… mindfulness might have been better if I had it before I got an acute phase, if that makes any sense. I might have had those tools in place before, that it might have prevented those feelings of falling.”*

CMR037

### 3.3. Theme 3: Practicalities of CMR Programme Participation

The final theme reflects on the practicalities of participating in the CMR programme and potential adaptation for future delivery.

#### 3.3.1. Subtheme 1: Challenges and Barriers to Participation

Participants experienced several challenges during the programme, identifying that participation required a level of vulnerability which they initially found uncomfortable but were able to overcome during the programme.

*“She really challenged us to open up… so perhaps after a meditation or an exercise, she would want to know what had been brought up and for us to share. And I found that very difficult. And I was a little bit taken aback by that on the first week. I hadn’t expected that.”*

CMR021

Some participants felt hesitant to share their personal experience as their symptoms were not as severe as those of others in the group or did not want to discourage others in earlier stages of CKD by revealing negative emotions after a successful transplant.

*“I felt that I couldn’t really say any of the bad things that I’ve experienced, because I was this sort of golden shining thing, having a successful transplant for lots of years.”*

CMR058

A number of participants experienced barriers to participating on the required date and time due to circumstances beyond their control, such as family emergencies and illness, while others had made significant efforts to take time off work to attend.

*“Personally I took holiday to make sure that I could fit it in and wouldn’t be distracted and I could focus on it one hundred percent. But yeah, obviously that’s a small barrier to that is having the ability.”*

CMR007

Several participants reflected on the challenge of making time to practice mindfulness; however, those who dedicated time to a regular practice expressed how beneficial this had been.

*“I think for me, at the beginning, the biggest barrier was implementing the practices from week to week and finding the time to do that, rather than having two hours set aside each week.”*

CMR044

#### 3.3.2. Subtheme 2: Experience of Online Delivery

Participants responded positively to the experience of online delivery, with most being familiar with Zoom software due to its prevalence during the COVID-19 pandemic. Most participants reflected that they would not have participated in a face-to-face programme due to ongoing shielding or the need to travel.

*“I guess I might have struggled a bit more with commitment if I’d to jump on a train and go into Edinburgh every couple of weeks. So I actually think it’s probably a useful thing. It didn’t restrict me in any way at all.”*

CMR034

Participants identified that participating from their homes was a safe environment for them, where they felt comfortable and most at ease.

*“You’re very safe in your own home. And you can mute or you can turn off the camera if you’re feeling a wee bit emotional.”*

CMR037

A small number of participants reported issues when accessing Zoom on their mobile phone rather than a laptop, and felt that holding a short session prior to the first CMR sessions might be beneficial for participants to practice using the technology. Closed captions were also made available for participants to use.

*“It was just the way I was trying to do it on the phone and I couldn’t see one of the buttons at the side, so it kept rejecting it. So when I looked at it on a laptop eventually, I sorted that out. But that’s just a technical issue.”*

CMR034

#### 3.3.3. Subtheme 3: Adaptations for Future Delivery for People Living with Kidney Disease

Participants suggested a range of adaptations to consider for future delivery of the CMR programme. A number of participants suggested that patients could be grouped into stages of kidney disease to enable more shared experience; however, other participants felt that they had much to learn from patients who were at different stages of kidney disease.

*“Maybe grouping people maybe would be perhaps a way that those who are stage five, pre-transplant or… I don’t know. But maybe.”*

CMR007

Participants expressed that a website or resource containing the course materials including presentation slides, meditations, and practices would be beneficial to revisit the programme content. Participants also felt they would prefer audio meditation practices recorded by their own teacher rather than a generic recording. A number of participants expressed an interest in the CMR resources being translated into other languages for those with English as a second language.

*“I would have thought some sort of website with all these materials and stuff like that, and short presentations on some of the themes that the course has given, would give people an opportunity to refresh their minds.”*

CMR034

Several participants identified that a clearer introduction to the content of the CMR, which defined the concepts and themes discussed within the programme, may have been beneficial, alongside an opportunity to ask questions and meet other members of their group, which may have enhanced their initial understanding of the programme.

*“As opposed to saying, we have a CMR course, would you like to do it? Or, we do compassionate mindfulness resilience. Would you like to do it? Those three words have very different meanings. And I think if they are… you know, maybe you do podcasts or whatever Kidney Care UK want to do, or however they feel. But it needs to be opened up so that people understand, this is what it’s about.”*

CMR026

## 4. Discussion

The qualitative analysis of this study aimed to explore participants’ experiences of the CMR programme, and provide further understanding of factors influencing the acceptability and suitability of the intervention within the wider scope of the feasibility study. Responses from participants have provided new insights into how a mindfulness intervention, such as the CMR programme, can be delivered online for people living with kidney disease within a group setting, identifying challenges and potential adaptions for future delivery. Analysis of interview data suggests that the intervention can be considered acceptable for this patient group, with all participants interviewed stating that taking part in the CMR programme had been a beneficial experience. While the qualitative findings have suggested that the four-session programme has provided useful mindfulness tools and techniques, longer-term support may be required to adequately support the mental wellbeing of people living with kidney disease, and earlier intervention may prove even more beneficial.

The themes and subthemes reported in this study are consistent with findings from previous studies exploring mindfulness interventions for people living with kidney disease. Common themes include participants’ improved ability to manage negative emotions and feel in control of their emotions [21], and reduced feelings of anxiety and depression [21] following participation in mindfulness interventions. Participants in previous studies reported improved communication with friends, family, and others [22], greater ability to feel self-compassion [23],, and improved sleep following mindfulness meditation [21], with the provision of recorded audio meditations identified as being beneficial for participants to continue their practice [24]. Participants in previous studies have also reported improvements in wellbeing [24], emotional regulation, and the ability to be mindful [25]. The benefits of participating in the CMR programme align with findings from studies undertaken with patients living with transplants, namely reduced feelings of depression and anxiety, improved sleep, and a better quality of life [20]. Findings from mindfulness studies for patient groups living with other chronic conditions have reported improved quality of life and mental wellbeing [40], and have identified the beneficial nature of shared patient experience and peer support, and the need for ongoing mindfulness support [41]. While the majority of previous studies delivered longer mindfulness programmes (8–12 weeks) in a face-to-face setting for people with kidney disease, the findings from the COSMIC study align with these results and provide evidence that shorter interventions such as the CMR programme, which are delivered online, have the potential to be beneficial for this patient population.

### Limitations

A limitation of the study is the relatively low response rate (n = 21, 32%) of participants who agreed to interview. Participants who volunteered for interview may have responded more positively to the mindfulness intervention and have been more motivated to discuss their experience. They may also have been in better health, and therefore able to participate in the interview, or may have had a pre-existing interest in mindfulness.

Researchers are aware that social desirability bias may be possible in regard to participants responses, and acknowledge this is a common issue within the realm of qualitative research [42]. The research team attempted to mitigate against this bias by using open-ended questioning, assuring participants of anonymity, building trust and rapport with the participants during the course of the study, and encouraging participants to share their true experiences and perspectives [43].

Much of the research on mindfulness and its benefits in health contexts has been conducted on predominantly white, middle-class, female populations, a trend reflected within the demographics of our study participants [44]. This underrepresentation of diverse ethnic and gender groups can lead to the generalization of findings that may not be applicable or effective for individuals from different backgrounds, and further work is required to address these issues when considering a larger randomised control trial of this intervention.

The analysis of participant interviews has identified the potential benefits of participating in an online group mindfulness intervention; however, the potential of ongoing support for patients should be considered, and further research is required to identify sustainable approaches to mental wellbeing support for this patient group. As this qualitative analysis was conducted as part of a single-group multi-methods feasibility study, a larger randomised clinical control trial, to include qualitative exploration of participant experience, is recommended to evaluate the efficacy of the intervention for this patient population.

## 5. Conclusions

In conclusion, the qualitative exploration of participant experience has demonstrated that the four-session online CMR programme can be considered an acceptable and suitable intervention for people living with kidney disease, providing beneficial tools and techniques to support the mental health and wellbeing of this patient group. Future research in this area would benefit from a definitive clinical trial, including a qualitative evaluation of the programme, to rigorously evaluate the efficacy of the intervention and establish if the CMR programme is a sustainable approach to support the mental health and wellbeing of people living with kidney disease.

## Figures and Tables

**Table 1:** Interview Participant Characteristics.

Characteristic	Participants Analysed (n = 19)
**Age (years)** n (%) Mean (SD)	19 (100%) 51.6 (8.34)
**Gender, n (%)** Female Male	14 (73.7%) 5 (26.3%)
**Ethnicity, n (%)** Black, African, Caribbean, or Black British—African White—Any other White background Black, African, Caribbean, or Black British—Caribbean White—English, Welsh, Scottish, Northern Irish or British Asian or Asian British—Indian White—Irish	1 (5.3%) 1 (5.3%) 2 (10.5%) 13 (68.4%) 1 (5.3%) 1 (5.3%)
**Marital status, n (%)** Divorced Married Widowed Single	2 (10.5%) 13 (68.4%) 1 (5.3%) 3 (5.3%)
**Highest educational level, n (%)** Level 1 Level 3 Level 4 + No qualifications Other	1 (5.3%) 1 (5.3%) 15 (78.9%) 1 (5.3%) 1 (5.3%)
**Years since diagnosis (years)** Mean (SD)	20.42 (11.33)
**Stage of CKD, n (%)** Post-transplant Stage 5 Stage 4	11 (57.9%) 4 (21.1%) 4 (21.1%)

**Table 2:** Thematic analysis themes and subthemes.

Theme	Subthemes
Experiences of the CMR programme that facilitated subjective benefit	Interest in mindfulness and previous experience
	Integrating mindfulness using techniques and practices to enhance awareness and compassion
	Continuing mindfulness practice
Participants’ lived and shared experiences	Psychological impact of kidney disease and continuing impact of COVID-19
	Shared experiences with other study participants
	Need for wellbeing support for people living with kidney disease
Practicalities of CMR programme participation	Challenges and barriers to participation
	Experience of online delivery
	Adaptations for future delivery for people living with kidney disease

## Data Availability

The data set for this qualitative study can be provided by contacting the authors of the paper.

## Supplementary Materials

The following supporting information can be downloaded at: https://www.mdpi.com/article/10.3390/healthcare11222926/s1, S1—Consolidated criteria for reporting qualitative research (COREQ) checklist. S2—Participant Interview Schedule.
