# Supplementary material for: Experiences of People with Kidney Disease Following the Implementation of the Compassionate Mindful Resilience Programme: Qualitative Findings from the COSMIC Study
Source: Healthcare (Basel). 2023 Nov 8;11(22):2926. doi: 10.3390/healthcare11222926 (PMC10671038; doi:10.3390/healthcare11222926)
Supplement: Supplementary file 1 [file healthcare-11-02926-s001.zip › S1.pdf]

Consolidated criteria for reporting qualitative studies (COREQ): 32-item checklist

Developed from:
Tong A, Sainsbury P, Craig J. Consolidated criteria for reporting qualitative research (COREQ): a 32-item checklist for interviews and focus groups. *International Journal for Quality in Health Care*. 2007. Volume 19, Number 6: pp. 349 – 357

**No. Item**	**Guide questions/description**	**Section #**
**Domain 1: Research team and reflexivity**		
Personal Characteristics		
1. Interviewer/facilitator	Which author/s conducted the interview or focus group?	Data Collection
2. Credentials	What were the researcher's credentials? E.g. PhD, MD	Recruitment
3. Occupation	What was their occupation at the time of the study?	Recruitment
4. Gender	Was the researcher male or female?	Recruitment
5. Experience and training	What experience or training did the researcher have?	Data Analysis
Relationship with participants		
6. Relationship established	Was a relationship established prior to study commencement?	Recruitment
7. Participant knowledge of the interviewer	What did the participants know about the researcher? e.g. personal goals, reasons for doing the research	Recruitment
8. Interviewer characteristics	What characteristics were reported about the interviewer/facilitator? e.g. Bias, assumptions, reasons and interests in the research topic	Recruitment

**Domain 2: study design**		
Theoretical framework		
9. Methodological orientation and Theory	What methodological orientation was stated to underpin the study? e.g. grounded theory, discourse analysis, ethnography, phenomenology, content analysis	Design
Participant selection		
10. Sampling	How were participants selected? e.g. purposive, convenience, consecutive, snowball	Recruitment
11. Method of approach	How were participants approached? e.g. face-to-face, telephone, mail, email	Recruitment
12. Sample size	How many participants were in the study?	Sample Size
13. Non-participation	How many people refused to participate or dropped out? Reasons?	Results
Setting		
14. Setting of data collection	Where was the data collected? e.g. home, clinic, workplace	Data Collection
15. Presence of non-participants	Was anyone else present besides the participants and researchers?	Data Collection
16. Description of sample	What are the important characteristics of the sample? e.g. demographic data, date	Recruitment
Data collection		
17. Interview guide	Were questions, prompts, guides provided by the authors? Was it pilot tested?	Data Collection
18. Repeat interviews	Were repeat interviews carried out? If yes, how many?	Data Collection
19. Audio/visual recording	Did the research use audio or visual recording to collect the data?	Data Analysis
20. Field notes	Were field notes made during and/or after the interview or focus group?	Data Collection
21. Duration	What was the duration of the interviews or focus group?	Data Collection
22. Data saturation	Was data saturation discussed?	Data Analysis
23. Transcripts returned	Were transcripts returned to participants for comment and/or correction?	Data Analysis
**Domain 3: analysis and findings**		
Data analysis		
24. Number of data coders	How many data coders coded the data?	Data Analysis

25. Description of the coding tree	Did authors provide a description of the coding tree?	Data Analysis
26. Derivation of themes	Were themes identified in advance or derived from the data?	Data Analysis
27. Software	What software, if applicable, was used to manage the data?	Data Analysis
28. Participant checking	Did participants provide feedback on the findings?	Data Analysis
Reporting		
29. Quotations presented	Were participant quotations presented to illustrate the themes/findings? Was each quotation identified? e.g. participant number	Results
30. Data and findings consistent	Was there consistency between the data presented and the findings?	Results
31. Clarity of major themes	Were major themes clearly presented in the findings?	Results
32. Clarity of minor themes	Is there a description of diverse cases or discussion of minor themes?	Results
