# Supplementary material for: Experiences of People with Kidney Disease Following the Implementation of the Compassionate Mindful Resilience Programme: Qualitative Findings from the COSMIC Study
Source: Healthcare (Basel). 2023 Nov 8;11(22):2926. doi: 10.3390/healthcare11222926 (PMC10671038; doi:10.3390/healthcare11222926)
Supplement: Supplementary file 1 [file healthcare-11-02926-s001.zip › S2.pdf]

Participant Interview Schedule

Introduction

Interviewer welcomes the participant.

Interviewer introduces themselves.

Interviewer provides an overview of the study, and why Queen's University Belfast, Kidney Care UK and Mindfulness UK are undertaking this research.

Interviewer talks through key points about the interview process - the interview is voluntary in nature, confidentiality, recording the interview, no right or wrong answers to the questions, all opinions and views will be appreciated.

Interviewers invites the participant to raise any questions/ concerns they have prior to beginning the interview.

Interview questions based on the RE-AIM QuEST evaluation framework.

Reach

Can you tell me a little about why you decided to take part in the Compassionate Mindful Resilience programme?

Have you any previous experience of mindfulness?

What were your expectations of the CMR course?

Effectiveness

Do you feel like taking part in the programme was beneficial for you?

- If so, in what ways?
- If not, why?

Was there anything you did not enjoy, or found challenging, about taking part in the programme?

Do you think the programme improved your ability to be mindful?

- If so, in what ways?
- If not, why do you think that is?

Do you think the inclusion of compassion within the programme was beneficial, and was there a good balance of mindfulness and compassion?

Do you think the programme could be beneficial for other people living with Chronic Kidney Disease?

- If so, in what ways?
- If not, why?

Can you suggest any ways that the programme could be made more effective for people with kidney disease?

Adoption

Do you feel that you were able to access information about the programme easily?

Did you experience any barriers in committing to taking part in the 4-week programme?

Were there any benefits you experienced that motivated you to continue to participate?

Were there any difficulties that made you feel less motivated to participate?

Implementation

How do you feel about the online delivery method of the programme?

Do you feel that the 2-hour weekly sessions over 4 weeks were manageable for you?

Could any changes be made to improve the experience of taking part in future?

How did you feel about completing the questionnaires/scales?

- Were they difficult/easy to complete?
- Was there anything you found that helped to complete them? (Researcher assistance/family input)

Maintenance

Did you practice at home using the audio meditations provided?

- If so, often?
- Did you feel the practices were too short/too long?

Do you think you will continue practicing mindfulness in the future?

Do you think a Mindfulness course, such as the CMR programme, would be a valuable asset for Kidney Care UK to offer as part of its programme of support services for people living with, or caring for, people with kidney disease?
